# Retraction: Blood biomarker discovery for Autism Spectrum Disorder: A proteomic analysis

**DOI:** 10.1371/journal.pone.0301648

**Published:** 2024-03-28

**Authors:** The PLOS ONE Editors

After publication of this article [1], concerns were raised about the analyses presented in the article and an inability to replicate the published results.

In response, the authors clarified that the correlation analysis reported in [1] included typically developing (TD) participants in error, whereas it should have only included participants with autism spectrum disorder (ASD). The TD controls were randomly assigned Total ADOS Scores of 1, 2, and 3 for the purposes of the analysis; ADOS scores were only collected for participants with ASD.

The *PLOS ONE* Editors retract this article because the correlation analyses and the conclusions centered on the panel of nine potential biomarkers are not reliable.

The authors are repeating the correlation analyses with the TD data excluded, and they stand by the proof-of-concept conclusion that proteomic analysis can identify a panel of blood biomarkers for ASD.

LH, MD, JL, DCG, and JAM agreed with the retraction. CS either did not respond directly or could not be reached.
